# Whole exome sequencing of patients who resolved Crohn’s disease and complex regional pain syndrome following treatment for paratuberculosis

**DOI:** 10.1186/s13099-019-0311-z

**Published:** 2019-06-20

**Authors:** J. Todd Kuenstner, Maher Kali, Christine Welch

**Affiliations:** 1 Department of Pathology, Temple University Hospital, 3401 N. Broad St., Philadelphia, PA 19140 USA; 2 CAMC Clinical Trials Center, 3100 MacCorkle Avenue S.E., Suite 806, Charleston, WV 25304 USA; 3 Outcomes Research, 3100 MacCorkle Avenue S.E., Suite 806, Charleston, WV 25304 USA

**Keywords:** *Mycobacterium avium* ssp. *paratuberculosis*, Crohn’s disease, Complex regional pain syndrome, Lymphangiomatosis, Type 1 diabetes mellitus, Genetic susceptibility, Single nucleotide polymorphism

## Abstract

**Background:**

A whole exome sequencing study was performed on an extended family including a patient with Crohn’s disease (CD) and a patient with complex regional pain syndrome (CRPS). The patient with CD and the patient with CRPS have experienced resolution of their disease following treatment for paratuberculosis. The study was performed in order to determine if there is an unusual mutation in this extended family that would explain the susceptibility to mycobacterial infection among many of the members.

**Results:**

We identified sets of rare single nucleotide polymorphisms (SNPs) that were shared among affected family members, including variants in two genes, IL15RA and CASP10, which have established roles in the immune response. In addition, the CD and CRPS patients were found to have heterozygous mutations in MBL2 and DDX58, mutations that have been associated with susceptibility to tuberculosis.

**Conclusions:**

The IL15RA and CASP10 variants may contribute to the disease symptoms exhibited in this family. The finding of SNPs associated with immune function supports a complementary role of infection and genetics in these diseases.

**Electronic supplementary material:**

The online version of this article (10.1186/s13099-019-0311-z) contains supplementary material, which is available to authorized users.

## Background and aims

Most research in Crohn’s disease (CD) has focused on genetics rather than the association of the disease with infection by *Mycobacterium avium* ssp. *paratuberculosis* (MAP) [1–4]. Over 140 genetic mutations have now been identified in CD [5]. These two areas of research are probably complementary because some of the genetic mutations, may indicate increased susceptibility to MAP infection. For example, the NOD2 mutation found in some CD patients is associated with susceptibility to MAP infection in cattle [6]. Furthermore, a 2009 study from China showed that patients with leprosy and patients with CD have higher frequencies of NOD2, TNFSF15 and IL12B mutations than healthy controls [7, 8]. A large meta-analysis genome-wide association study (GWAS) concluded that there is considerable overlap between the susceptibility loci for inflammatory bowel disease (IBD) and mycobacterial infection [9].

In a recent case series report, four patients with multiple diseases of unknown etiology (cases 1–4) and one healthy individual (case 5) were found to have evidence of infection by MAP including positive blood cultures (except in case 4 which had positive MAP antibodies). Two of the cases (case 1 with CD and asthma and case 2 with CRPS, hypothyroidism and Raynaud’s phenomenon) were treated with a combination of anti-MAP antibiotics and ultraviolet blood irradiation (UVBI) with resolution of the diseases and inability to culture MAP in post treatment blood samples. These case reports of patients with MAP infections provide supportive evidence of a pathogenic role of MAP in humans [10].

**Fig. 1:**
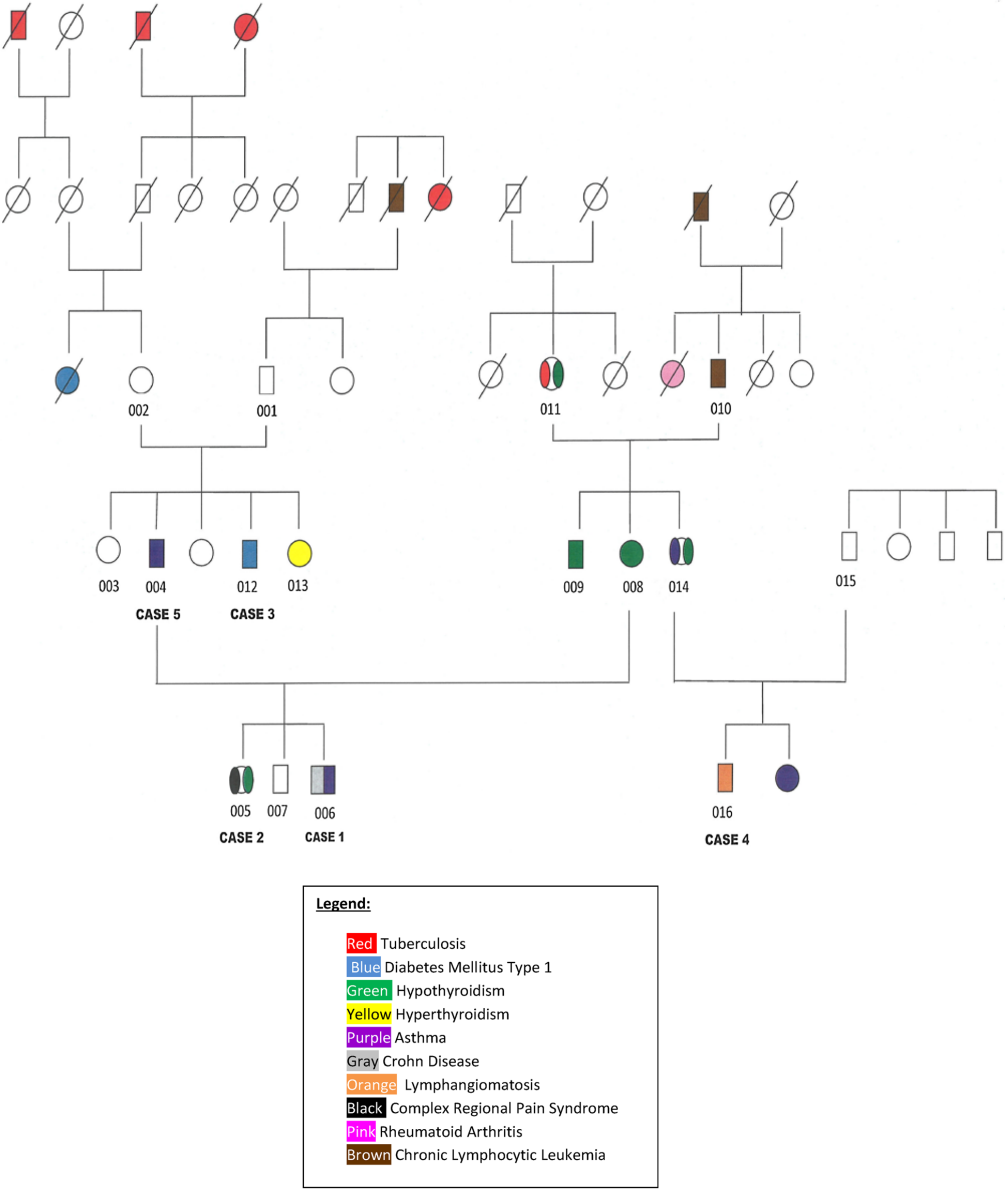
Family pedigree summarizing the history of mycobacterial infection and other diseases in cases 1 through 5 and in additional family members. Each study subject number (001-016) appears beneath the corresponding subject. Cases 1–5 are 006, 005, 012, 016 and 004, respectively. This figure previously appeared in reference 10 and is used with permission of the publisher

The role of genetic mutations which might confer increased susceptibility to mycobacterial infection and immune disorders was investigated by whole exome analysis in this family with an extensive history of mycobacterial infection and diseases traditionally considered autoimmune (see Fig. 1).

## Results

We recruited members of a single family where individuals were affected by asthma, CD, hypothyroidism and CRPS and performed whole exome sequencing on 10 members of the family (see Fig. 1 and Table 1).

**Table 1:** Whole exome read and alignment data

Sample ID	Total reads pass filter	Total aligned reads	Percent aligned reads	SNP percent found in dbSNP	Mean coverage
315-001-003	85,396,980	78,226,223	91.6	98.8	41.1X
315-001-004	116,661,046	114,816,183	98.4	98.8	58.9X
315-001-005	111,609,738	109,886,187	98.5	98.9	57.3X
315-001-006	140,466,506	137,765,626	98.1	98.9	72.8X
315-001-008	133,788,894	126,633,454	94.7	99.2	65.8X
315-001-009	119,135,414	115,697,404	97.1	98.8	57.6X
315-001-010	114,281,892	111,467,858	97.5	99	57.2X
315-001-011	133,211,626	130,104,251	97.7	98.7	72.9X
315-001-012	139,647,612	136,534,857	97.8	98.8	78X
315-001-014	170,815,864	168,254,316	98.5	98.9	99.5X

We searched for variants present in any of five CD susceptibility genes (ATG16L1, IL23R, CARD9, NOD2, and PTGER4) in any affected family member using models 1, 2 and 5 in Table 2. We detected no variants that met the criteria in any affected family member under any of the three models. Removal of the requirement for absence in the normal family member revealed two variants which were found under all three models: (1) ATG16L1 rs2241880, a missense variant (Thr318Ala), has an allele frequency of 0.456 and is rated as benign by Polyphen2 and tolerated by SIFT and (2) IL23R rs7530511 a missense variant (Leu310Gln) has a frequency of 0.166 and is also rated as benign.

**Table 2:** Genetic models and filtration chains

Inheritance model	1	2	3	4	5	6
	Auto dominant	Auto dominant	Auto recessive	Auto recessive	Auto dominant	Auto recessive
	Paternal affected	Maternal affected	Paternal affected	Maternal affected	Myco susceptible	Myco susceptible
Reference genome	38	38	38	38	38	38
Sequence ontology (variant locations)	Coding + SA/SD	Coding + SA/SD	Coding + SA/SD	Coding + SA/SD	Coding + SA/SD	Coding + SA/SD
Allele annotation source	Ensembl Genes90	Ensembl Genes90	Ensembl Genes90	Ensembl Genes90	Ensembl Genes90	Ensembl Genes90
Minor allele frequency	Less than 0.05 or missing	Less than 0.05 or missing	Less than 0.05 or missing	Less than 0.05 or missing	Less than 0.05 or missing	Less than 0.05 or missing
Affected members	4, 5, 6, 12	5, 6, 8, 9, 11, 14	4, 5, 6, 12	5, 6, 8, 9, 11, 14	4, 5, 6, 12	4, 5, 6, 12
Unaffected member	3	10	3	10	10	10
Sharing filter (number of affected members)	all N (=4)	all N (=6)	all N (=4)	all N (=6)	all N (=4)	all N (=4)
Zygosity filter	Homozygous minor allele + heterozygotes	Homozygous minor allele + heterozygotes	Homozygous minor allele	Homozygous minor allele	Homozygous minor allele + heterozygotes	Homozygous minor allele + heterozygotes
Sequence read depth filter	10 or greater	10 or greater	10 or greater	10 or greater	10 or greater	10 or greater
Polyphen status (probably or possibly damaging mutation) or not called by Polyphen	Possibly or probably or not called	Possibly or probably or not called	Possibly or probably or not called	Possibly or probably or not called	Possibly or probably or not called	Possibly or probably or not called

Inheritance model	7	8	9	10	11	12
	Auto dominant	Auto dominant	Auto recessive	Auto Recessive	Auto dominant	Auto recessive
	Paternal affected	Maternal affected	Paternal affected	Maternal affected	Myco susceptible	Myco susceptible
Reference genome	38	38	38	38	38	38
Sequence ontology (variant locations)	Coding + SA/SD	Coding + SA/SD	Coding + SA/SD	Coding + SA/SD	Coding + SA/SD	Coding + SA/SD
Allele annotation source	Ensembl Genes90	Ensembl Genes90	Ensembl Genes90	Ensembl Genes90	Ensembl Genes90	Ensembl Genes90
Minor allele frequency from 1 K genomes	Less than 0.05 or missing	Less than 0.05 or missing	Less than 0.05 or missing	Less than 0.05 or missing	Less than 0.05 or missing	Less than 0.05 or missing
Affected members	4, 5, 6, 12	5, 6, 8, 9, 11, 14	4, 5, 6, 12	5, 6, 8, 9, 11, 14	4, 5, 6, 12	4, 5, 6, 12
Unaffected member	3	10	3	10	10	10
Sharing filter (number of affected members)	N − 1(=3)	N − 1(=5)	N − 1(=3)	N − 1(=5)	N − 1(=3)	N − 1(=3)
Zygosity filter	Homozygous minor allele + heterozygotes	Homozygous minor allele + heterozygotes	Homozygous minor allele + heterozygotes	Homozygous minor allele + heterozygotes	Homozygous minor allele + heterozygotes	Homozygous minor allele + heterozygotes
Sequence read depth filter	10 or greater	10 or greater	10 or greater	10 or greater	10 or greater	10 or greater
Polyphen status (probably or Possibly damaging mutation) or not called by Polyphen	Possibly or probably or not called	Possibly or probably or not called	Possibly or probably or not called	Possibly or probably or not called	Possibly or probably or not called	Possibly or probably or not called

Shared variants were selected under 12 different models. Each row describes the criteria for each filtration step while column indicates the genetic model. Splice Acceptor (SA), Splice Donor (SD). Minor allele frequencies were based on 1000 Genomes data

We also searched our variant data sets for established tuberculosis susceptibility genes and loci that had variants present in any affected family member using three autosomal dominant models. In this analysis, we used the filter chains for models 1, 2 and 5 in Table 2. with the following modifications: (1) The minor allele frequency and Polyphen filters were removed, (2) Sequence ontology was expanded to include 5′ and 3′ UTRs, and (3) A filter that selected for variants present in DDX58, IL10, IL12B, MBL2, SLC11A1 and two Tuberculin Skin Test Reactivity loci (TST1 11p14-15, TST2, 5p15) was added [11–14]. Under the modified versions of models 1, 2 and 5, we identified 12, 13 and 10 variants, respectively. Gene names, variant ID numbers, predicted pathogenicity and variant allele frequencies are provided in Additional file 1: Appendix 1.

Next, we investigated whether a genetic variant with pleiotropic effects could cause clinically different presentations in affected family members and whether a single variant might underlie these presentations. In order to identify variants that could contribute to the disease state, we performed discrete filtration under three autosomal dominant (AD) models and three autosomal recessive (AR) models found in Table 2. In models 1 (AD) and 3 (AR) we searched for variants that were shared by those who were affected by asthma (patient 004), CRPS (patient 005), CD (patient 006) and diabetes (patient 012) and absent from a paternal normal (patient 003). In models 2 (AD) and 4 (AR), we required that the disease variant was present in patients with asthma (patient 006), CD (patient 006), CRPS (patient 005), or hypothyroidism (patients 005, 008, 009, 011 and 014) and absent in a normal maternal relative (patient 010). We also sought shared variants under models based on mycobacterial infection of family members 004, 005, 006 and 012 under an autosomal dominant model (#5) and an autosomal recessive model (#6).

We constructed discrete filtration chains using VarSeq software in order to identify shared genetic variants (single nucleotide variants (SNVs) and small indels); filter sets for each chain are shown in Table 2. The number of variants retained after each step in the filtration chain is shown in Table 3. Under models 1, 2, 3, 4, 5, 6, we identified 57, 56, 1, 14, 41 and 1 variants, respectively, that were shared in all affected members. Since the disease state of some affected members may stem from locus heterogeneity (i.e. different genetic etiologies), we repeated the analysis with the six additional filtration chains (models 7–12 in Table 2) where we required N − 1 members of the affected group to share a variant instead of all affected members. As expected, reduction of stringency in the shared variant step increased the numbers of variants for most of the models (Table 3).

**Table 3:** Discrete filtration

Inheritance model	1	2	3	4	5	6
Sequence ontology	24,623	26,950	24,623	26,950	29,197	29,612
European allele frequency ≤ 0.05 or missing	11,024	12,864	3144	13,899	15,089	15,592
Variants shared among affected members	903	655	974	1432	1869	1500
Variants absent from normal or not called	110	96	2	16	73	2
Sequence read depth ≥ 10 for all patients	95	89	1	15	68	1
Polyphen = possibly or probably damaging or	57	56	1	14	41	1

Inheritance model	7	8	9	10	11	12
Sequence ontology	24,623	26,950	24,623	26,949	29,197	29,612
European Allele ≤ frequency 0.05 or missing	11,024	12,864	3144	13,898	15,089	15,592
Variants shared among affected members	2228	1213	1012	1636	3239	1816
Variants absent from normal or not called	577	292	6	34	353	14
Sequence read depth ≥ 10 for all patients	142	111	2	17	73	3
Polyphen = possibly or probably damaging or	91	74	1	16	45	2

We screened for relevant variants using twelve different genetic models as described in Table 2. Rows provide the number of variants following application of each filtering criterion

In order to assess the significance of the shared variants from models 1-12, we collapsed variants into unique genes (gene symbols), entered each symbol into the GeneCards and MalaCards databases [15, 16] and generated lists of shared genes and associated disease states (Additional file 1: Appendix 2). We then searched these lists for the term autoimmune or interleukin. Two variants were identified in these searches: (1) a variant in Interleukin 15 receptor alpha (IL15RA, rs8177777) is shared by all affected members under models 1, 5, 7 and 11. The allele frequency of rs8177777 is 0.01 in the ExAC database [17] and 0.0082 in the 1000 Genomes database [18]. All affected members are G/A heterozygotes. IL15RA encodes the alpha subunit of a cytokine receptor which binds interleukin 15 (IL15) and is critical to the early immune response to microbial infections. The variant causes a missense substitution of alanine to valine at position 97 (Ala97Val) in IL15RA isoform X1. MutationTaster, a predictor of missense functional consequences rated this variant as tolerated [19] (2) Using models 2 and 8, all affected members were heterozygous for SNP rs17860405 which leads to a tyrosine to cysteine missense at position 446 (Tyr446Cys) in the caspase 10 protease (CASP10). Its allele frequency is 0.0295 in the ExAC database and 0.0128 in the 1000 Genomes database. Polyphen and SIFT rate this variant as possibly damaging [20] and tolerated [21], respectively.

Finally, we searched for whole exome variant sets for variants in ORMDL3 (mutations observed in CD and asthma) under four models: autosomal dominant with affected parental members (model #1), autosomal recessive with affected parental members (model #2), autosomal dominant with mycobacterial susceptible members (model #5), and autosomal recessive with mycobacterial susceptible members (model #6). All four of these models invoked 4, 5, 6 and 12 as affected subjects.

In each case, the filtration chain was modified to resemble previous searches for named MS genes that we previously did. These changes included (a) expanding the sequencing ontology to include 5′ and 3′ UTRs and synonymous missense variants, (b) removing the allele frequency filter (so that alleles with any frequency would be allowed), (c) removing the requirement for sharing variants among the four affected members (4, 5, 6 and 12) such that the presence of a variant in any of the affected members would be allowed, and (d) removing the Polyphen pathogenicity filter. The filter which required a read depth of 10 or more for each variant call was retained. The sequence ontology filter excluded intronic and intergenic variants as did the named gene searches. In spite of the expanded filtration design, no ORMDL3 variants in subjects 4, 5, 6 and 12 were found under any of the models.

## Discussion

In this study, none of the affected patients were found to have any of 5 common mutations in CD. The CD and CRPS patients were found to have heterozygous mutations in MBL2 and DDX58, mutations that have been associated with susceptibility to tuberculosis.

In addition, we identified several rare genetic variants which were shared among affected members under twelve genetic models. A few variants were located in genes with known roles in the immune response (CASP10 and IL15RA).

For the cases in this study, the heterozygous combination of mutations of MBL2 and DDX58 may indicate increased susceptibility to mycobacterial infection. However, this and the previous study of this extended family suggests that the more important factor in the diseases of these hosts was MAP bacteremia and the presence and absence of MAP corresponded to the presence and resolution of several diseases. Moreover, a much greater percentage of patients with CD have evidence of MAP infection than those with any single SNP identified in this cohort of patients or any other cohort of CD patients also suggesting that MAP infection plays a more constant and important role in CD than the genetic constitution of the affected patients.

## Conclusions

The ever expanding number of genetic mutations associated with CD (over 140 susceptibility loci in 2014) [5], the increasing incidence and prevalence of CD [22], the corresponding increasing prevalence of MAP infection in cattle [23], and the recently published case series report with supportive evidence of the pathogenicity of MAP in humans [10] implicate MAP infection as an important candidate trigger of CD. Controlled clinical trials using a combination of UVBI and antibiotics to determine whether clearance of MAP bacteremia results in CD resolution in other patients are indicated.

## Methods

Patient recruitment and confidentiality: 16 family members of an extended family with multiple diseases of unknown etiology were recruited for the study following review and approval by the Charleston Area Medical Center Institutional Review Board (Institutional Review Board #1,997,444, April 29, 2014). Recruitment was conducted by the Charleston Area Medical Center Clinical Research Institute. Subjects were assigned a continuously-running study identification number to maintain confidentiality of records. Only de-identified data was shared with genomic data analysts. Subject data records were kept in a locked filing cabinet. Electronic subject data was maintained on password protected computers. All subject data were kept in strict confidence.

Genomic DNA isolation and whole exome sequencing: Buccal samples were collected from 15 of the 16 consented family members (subject 016 (case 4) did not submit a buccal swab) using a protocol designed for genomic DNA isolation. Each participant was asked not to consume any food or drink in the 30 min prior to sample collection and collected their own swab by placing the swab inside their mouth and scraping it firmly against the inside of each cheek 6 times. Buccal samples were then air-dried for at least 2 h after collection. After drying, the swab head was ejected into a sterile 2 ml screw cap microfuge tube which was labeled with patient study number. These samples were then shipped overnight in batches at room temperature to the Marshall University Genomics Core Facility (Huntington, WV) where the genomic analysis was performed.

Genomic DNA was extracted from buccal samples from fifteen patients using a QiaAmp DNA Mini kit (Qiagen, Germantown, MD) using the method described by the manufacturer and quantified Qubit fluorimetry (ThermoFisher Scientific, Waltham, MA). A_260/280_ absorbance ratios were in the 1.70 to 2.00 range for all samples. Indexed whole exome libraries were prepared from 50 ng of genomic DNA using Nextera Whole Exome enrichment kits (Illumina Inc., San Diego, California). Library yields were quantified by Qubit fluorimetry and insert sizes were determined by Agilent Bioanalyzer electrophoretic analysis. Insert sizes ranged from 273 bp to 700 bp. The resulting fifteen whole exome libraries were clustered on an Illumina cBot cluster station and sequenced in a 2 × 50 paired end strategy on an Illumina HiSeq 1500 running in the high output mode. Whole exome sequence data was analyzed using the BWA Enrichment application on Illumina Base Space. For the 10 libraries used in the filtration analysis described below, total reads/library ranged from 85,396,980 to 170,815,864. Percent aligned reads ranged from 91.6 to 98.8%. Alignments identified between 99.1 and 99.3% of SNPs found in dbSNP and between 90.9 and 93.6% of indels found in dbSNP. The mean coverage depth ranged from 41.1X to 107.1X. A summary of the whole exome read and alignment data appears in Table 1.

Reads were aligned to GRCh38 using Bowtie v2.0.1 [24]. Small variants (SNPs and indels) were identified using Samtools v0.1.18 [25] and output as VCF files. We filtered for potential causal variants under six different Mendelian models using VarSeq software v1.5.0 (Golden Helix, Bozeman MT). In general, VarSeq filtration chains were designed to identify low frequency variants that encoded pathogenic changes shared by all affected family members under the specified Mendelian model. Each chain contained the following filters: (1) A sequence ontology filter classified and collected variants that were frameshift, missense, splice acceptor, splice donor, splice region variant, initiator codon, stop gain, stop loss, stop-retained mutations, or in-frame deletions or insertions. We excluded variants that led to synonymous codon changes and variants that were located in intergenic, intronic and 5′ and 3′ UTR regions. (2) An allele frequency filter retained variants whose frequency was ≤ 5%. (3) A zygosity filter retained heterozygotes or variant homozygotes in the autosomal dominant mode or variant homozygotes in the autosomal recessive mode. (4) A shared variant filter retained variants that were common to all of the affected members or an N − 1 subset. (5) Variants present in an unaffected member were excluded. (6) A sequence read depth filter retained variants whose read counts were ≥ 10. (7) A pathogenicity filter retained variants scored as either possibly or probably damaging by Polyphen-2 (or unknown in the case of noncoding variants). Models 1–12 were built and named by Donald Primerano of Marshall University. Specific filters for each model are summarized in Table 2.

## Additional files

**Additional file 1.** Variants present in selected mycobacterial susceptibility loci.

**Additional file 2.** Search for shared genes and associated disease states in this extended family.

## Data Availability

Not applicable.

## Abbreviations

MAP: *Mycobacterium avium* ssp. *paratuberculosis*
UVBI: Ultraviolet blood irradiation using the B and C regions of the ultraviolet light spectrum
CD: Crohn’s disease
CRPS: complex regional pain syndrome
SNPs: single nucleotide polymphisms
SNVs: single nucleotide variants
IBD: inflammatory bowel disease
GWAS: genome wide association study
AD: autosomal dominant
AR: autosomal recessive
SA: splice acceptor
SD: splice donor
