# Supplementary material for: Whole exome sequencing of patients who resolved Crohn’s disease and complex regional pain syndrome following treatment for paratuberculosis
Source: Gut Pathog. 2019 Jun 20;11:34. doi: 10.1186/s13099-019-0311-z (PMC6587279; doi:10.1186/s13099-019-0311-z)
Supplement: Supplementary file 1 — Additional file 1. Variants present in selected mycobacterial susceptibility loci. [file 13099_2019_311_MOESM1_ESM.docx]

Appendix 1. Variants present in selected mycobacterial susceptibility loci
